# Supplementary material for: Localized and global representation of prior value, sensory evidence, and choice in male mouse cerebral cortex
Source: Nat Commun. 2024 May 22;15:4071. doi: 10.1038/s41467-024-48338-6 (PMC11111702; doi:10.1038/s41467-024-48338-6)
Supplement: Supplementary file 3 — Reporting Summary [file 41467_2024_48338_MOESM3_ESM.pdf]

nature portfolio

Corresponding author(s): Akihiro Funamizu

Last updated by author(s): Mar 26, 2024

Reporting Summary

Nature Portfolio wishes to improve the reproducibility of the work that we publish. This form provides structure for consistency and transparency in reporting. For further information on Nature Portfolio policies, see our Editorial Policies and the Editorial Policy Checklist.

Statistics

For all statistical analyses, confirm that the following items are present in the figure legend, table legend, main text, or Methods section.

n/a	Confirmed	
☐	☒	The exact sample size (*n*) for each experimental group/condition, given as a discrete number and unit of measurement
☐	☒	A statement on whether measurements were taken from distinct samples or whether the same sample was measured repeatedly
☐	☒	The statistical test(s) used AND whether they are one- or two-sided<br>*Only common tests should be described solely by name; describe more complex techniques in the Methods section.*
☐	☒	A description of all covariates tested
☐	☒	A description of any assumptions or corrections, such as tests of normality and adjustment for multiple comparisons
☐	☒	A full description of the statistical parameters including central tendency (e.g. means) or other basic estimates (e.g. regression coefficient) AND variation (e.g. standard deviation) or associated estimates of uncertainty (e.g. confidence intervals)
☐	☒	For null hypothesis testing, the test statistic (e.g. *F*, *t*, *r*) with confidence intervals, effect sizes, degrees of freedom and *P* value noted<br>*Give P values as exact values whenever suitable.*
☒	☐	For Bayesian analysis, information on the choice of priors and Markov chain Monte Carlo settings
☒	☐	For hierarchical and complex designs, identification of the appropriate level for tests and full reporting of outcomes
☒	☐	Estimates of effect sizes (e.g. Cohen's *d*, Pearson's *r*), indicating how they were calculated

Our web collection on statistics for biologists contains articles on many of the points above.

Software and code

Policy information about availability of computer code

Data collection	MATLAB 2016b (Bpod r0.5 framework; https://sanworks.io)<br>OpenEphys (Neuropixels PXI)
Data analysis	Codes which support findings in our paper are available on Github [https://github.com/funamizu-lab/Ishizu_et_al_2023] and deposited on Zenodo [https://doi.org/10.5281/zenodo.10881334]. We used following softwares for data analyses:<br>MATLAB 2021a (Behavioral and neural data analysis, glmnet (https://glmnet.stanford.edu/index.html))<br>MATLAB 2020a (Kilosort3; https://github.com/MouseLand/Kilosort)<br>Python Anaconda3 (Phy; https://github.com/cortex-lab/phy)

For manuscripts utilizing custom algorithms or software that are central to the research but not yet described in published literature, software must be made available to editors and reviewers. We strongly encourage code deposition in a community repository (e.g. GitHub). See the Nature Portfolio guidelines for submitting code & software for further information.

Data

Policy information about availability of data

All manuscripts must include a data availability statement. This statement should provide the following information, where applicable:

- Accession codes, unique identifiers, or web links for publicly available datasets
- A description of any restrictions on data availability
- For clinical datasets or third party data, please ensure that the statement adheres to our policy

The Spike data generated in this study including from the posterior parietal cortex and Source data are deposited in figshare [https://doi.org/10.6084/m9.figshare.24319705]. The raw electrophysiological data, which are not deposited because of the large data size (> 1 TB), are available from the corresponding author upon request.
The lists of our dataset are below:
Behavioral data:8 mice in 122 sessions (mouse i01-i08: 23, 12, 11, 21, 20, 20, 10, 5 sessions).
Electrophysiolosical data with Neuropixels 1.0:
Auditory cortex:7 mice in 29 sessions (mouse i01 – i08: 5, 1, 0, 4, 7, 7, 4, 1 sessions)
Secondary motor cortex: 8 mice in 34 sessions (6, 6, 6, 6, 3, 4, 1, 2 sessions)
Medial prefrontal cortex: 8 mice in 34 sessions (8, 5, 5, 3, 3, 4, 4,2 sessions)
Posterior parietal cortex: 6 mice in 32 sessions (7, 1, 0, 8, 7, 5, 4, 0 sessions; 7 sessions were simultaneously recorded with AC)

Research involving human participants, their data, or biological material

Policy information about studies with human participants or human data. See also policy information about sex, gender (identity/presentation), and sexual orientation and race, ethnicity and racism.

Reporting on sex and gender	N/A
Reporting on race, ethnicity, or other socially relevant groupings	N/A
Population characteristics	N/A
Recruitment	N/A
Ethics oversight	N/A

Note that full information on the approval of the study protocol must also be provided in the manuscript.

Field-specific reporting

Please select the one below that is the best fit for your research. If you are not sure, read the appropriate sections before making your selection.

☒ Life sciences ☐ Behavioural & social sciences ☐ Ecological, evolutionary & environmental sciences

For a reference copy of the document with all sections, see nature.com/documents/nr-reporting-summary-flat.pdf

Life sciences study design

All studies must disclose on these points even when the disclosure is negative.

Sample size	Data were sampled from 8 male mice. A sample-size calculation was not performed prior to the experiment, but we determined that the number of samples was sufficient to obtain the results as follow:<br>We measured the behavior of mice in 122 sessions from 8 mice, over 70,000 trials in total. In the analysis of animal behavior, we found that the behavioral model with reinforcement learning succeeded in generating the choice behavior of mice (Fig. 2).<br>For the analysis of neural activity, we measured the activity from around 30 sessions each from AC, M2, and mPFC (AC: 29, M2: 34, mPFC: 34 sessions), and collected more than 3,800 cells from each brain area (AC: 5,994, M2: 3854, mPFC: 9503). The number of neurons in this study is larger than conventional physiological experiments. Also, the number of neurons is larger than the 96 conditions that appear during our behavioral task (sound length (2) x sound frequency (6) x choice (2) x outcome (2) x reward size (2) = 96). We found that the neural activity in this study could encode and decode the task parameters (Fig. 3-8).
Data exclusions	Sessions in which the mouse did not achieve proper tone-frequency discrimination choices were excluded from further analyses. The definition of data exclusions and the number of excluded sessions were reported in Methods.
Replication	Reproducibility was confirmed by having 8 independent animals. The behavioral results in each mouse were consistent as shown in Supplementary Fig.1. The neural activity was also measured from 8 independent animals from both brain hemispheres. In each target brain area, the measurements were repeated between 1 and 4 times. The results of neural activity in each mouse were shown in Supplementary Fig. 12.

Randomization	This study had no comparisons among different groups of animals. The cross validation in this study was repeated 10 to 1000 times to reduce noise from randomization.
Blinding	This study had no explicit control groups. All experimental controls were within animals.

Reporting for specific materials, systems and methods

We require information from authors about some types of materials, experimental systems and methods used in many studies. Here, indicate whether each material, system or method listed is relevant to your study. If you are not sure if a list item applies to your research, read the appropriate section before selecting a response.

Materials & experimental systems

n/a	Involved in the study
☒	☐ Antibodies
☒	☐ Eukaryotic cell lines
☒	☐ Palaeontology and archaeology
☐	☒ Animals and other organisms
☒	☐ Clinical data
☒	☐ Dual use research of concern
☒	☐ Plants

Methods

n/a	Involved in the study
☒	☐ ChIP-seq
☒	☐ Flow cytometry
☒	☐ MRI-based neuroimaging

Animals and other research organisms

Policy information about studies involving animals; ARRIVE guidelines recommended for reporting animal research, and Sex and Gender in Research

Laboratory animals	We used 8 male CBA/J mice (Charles River, Japan), 8 to 13 weeks of age at the start of behavioral training. Mice were housed in a temperature-controlled room with the ambient temperature of 26.6 degrees Celsius with the average humidity of 23.3%.
Wild animals	No wild animals.
Reporting on sex	All animals were male. Many systems neuroscience studies, especially in the field of decision making, have long been used male rodents. To compare and advance the knowledge from previous studies, we designed our experiments with male mice. However, it is extremely important to examine the sex differences of neural circuits in decision making. We indicated this in Discussion as the limitation of our study and as for future experiments.
Field-collected samples	No field-collected samples.
Ethics oversight	All animal procedures were approved by the Animal Care and Use Committee at the Institute for Quantitative Biosciences (IQB), University of Tokyo.

Note that full information on the approval of the study protocol must also be provided in the manuscript.
